# Network propagation for GWAS analysis: a practical guide to leveraging molecular networks for disease gene discovery

**DOI:** 10.1093/bib/bbae014

**Published:** 2024-02-10

**Authors:** Giovanni Visonà, Emmanuelle Bouzigon, Florence Demenais, Gabriele Schweikert

**Affiliations:** Empirical Inference, Max-Planck Institute for Intelligent Systems, Tübingen 72076, Germany; Université Paris Cité, INSERM, UMRS-1124, Paris F-75006, France; Université Paris Cité, INSERM, UMRS-1124, Paris F-75006, France; School of Life Sciences, University of Dundee, Dundee DD1 5EH, Scotland, UK

**Keywords:** GWAS, network propagation, molecular network, disease gene

## Abstract

**Motivation:**

Genome-wide association studies (GWAS) have enabled large-scale analysis of the role of genetic variants in human disease. Despite impressive methodological advances, subsequent clinical interpretation and application remains challenging when GWAS suffer from a lack of statistical power. In recent years, however, the use of information diffusion algorithms with molecular networks has led to fruitful insights on disease genes.

**Results:**

We present an overview of the design choices and pitfalls that prove crucial in the application of network propagation methods to GWAS summary statistics. We highlight general trends from the literature, and present benchmark experiments to expand on these insights selecting as case study three diseases and five molecular networks. We verify that the use of gene-level scores based on GWAS *P*-values offers advantages over the selection of a set of ‘seed’ disease genes not weighted by the associated *P*-values if the GWAS summary statistics are of sufficient quality. Beyond that, the size and the density of the networks prove to be important factors for consideration. Finally, we explore several ensemble methods and show that combining multiple networks may improve the network propagation approach.

Identifying genes causally connected to disease is crucial for understanding disease aetiology and designing novel therapies. In some cases, human diseases are causally linked to a single gene. For example, mutations in the HTT gene, which encodes a protein called *huntingtin*, cause Huntington’s disease [1]. Many other diseases, such as Parkinson’s or Alzheimer’s disease, and several types of cancer, result from many genetic and environmental factors. Moreover, different combinations of genetic factors may cause the same or a related disease phenotype, which is due to genetic heterogeneity. In these cases, affected individuals exhibit the same or related disease phenotype, but the complex genetic causes may vary significantly between individuals. In addition, sequence variants that contribute to the disease phenotype in one individual with a specific genomic background may also be found in the healthy population.

In some instances, contributions from individual genetic variants do not simply add up, but combine in complex epistatic patterns [2, 3]. In general, multifactorial diseases are linked to multiple risk genes that influence disease occurrence in a complex manner [4].

GWA studies, paired with reference panels such as those provided by the 1000 Genomes Project Consortium [5] or the Haplotype Reference Consortium [6], can efficiently analyse up to $\sim\!\! 10^{7}$ locations across the genome for their contribution to a pathological phenotype, opening the way for unbiased large-scale discovery of disease genes. The genetic variants studied, most commonly single nucleotide polymorphisms (SNPs) and copy number variants (CNVs), that surpass a chosen significance threshold are subsequently analysed to identify disease-causing genes.

As GWA studies involve simultaneous statistical tests for numerous variants, it is likely that the compared groups (healthy vs diseased) will appear to differ in at least some of them by chance. Therefore, multiple hypothesis testing corrections have to be applied to prevent erroneous inferences. Typically, this requires a strict significance threshold for individual comparisons, possibly leading to loci being missed [7]. In particular, genetic variants with a low effect size or that interact with other variants are difficult to identify, while the variants detected by this approach typically have modest effect sizes and do not fully account for disease heritability. The analysis is further complicated by the fact that genetic variants are, in general, not independent of each other, due to factors such as different rates of genetic recombination and population structure. The resulting non-random associations between alleles (Linkage Disequilibrium, LD) introduces additional challenges in the analysis of population-level variants.

It is thus difficult to separate causal variants from the background noise of sequence variation, and large test populations are required to overcome a lack of power in the analysis of multifactorial diseases.

On the other hand, the polygenicity of a disease also offers opportunities; specifically, we may expect that genes involved in a specific phenotype might organize into known connected groupings or pathways. These network associations present an additional source of information to inference based on GWAS summary statistics.

Several approaches have been proposed to leverage this additional layer of biological knowledge. In this work, we focus on the challenges involved with the application of network propagation methods [8] to GWAS summary statistics. In this context, we employ *network propagation* as an umbrella term that gathers mathematical formalisms based on random walks (RWs) or information diffusion applied to graphs in which the nodes represent the genes and the edges highlight biological connections. We focus on GWAS summary statistics because this type of biological data can be easily shared without incurring the challenges of storing and publishing sensitive biological data.

With this work, we aim to provide insights into the steps that researchers have to implement to employ network propagation methodologies. The analysis procedure offers many opportunities for the implementation of suitable inductive biases. Firstly, the processing of the GWAS data and the subsequent aggregation of the SNP-level *P*-values can reflect different relationships between genetic variants and protein coding genes, based on 3D structure, genetic proximity, and functional relationships. The choice of molecular network also influences the type of information used to complement the GWAS summary statistics. Finally, the specific propagation algorithm can be selected and tuned to focus on different aspects of the genetic pathways of disease. A schematic representation of the aforementioned steps can be found in Figure 1. We will present insights from the published literature on these design choices, and then we will showcase a small case study where we apply a simple network propagation scheme to five selected networks to analyse GWAS results from three diseases.

**Figure 1:**
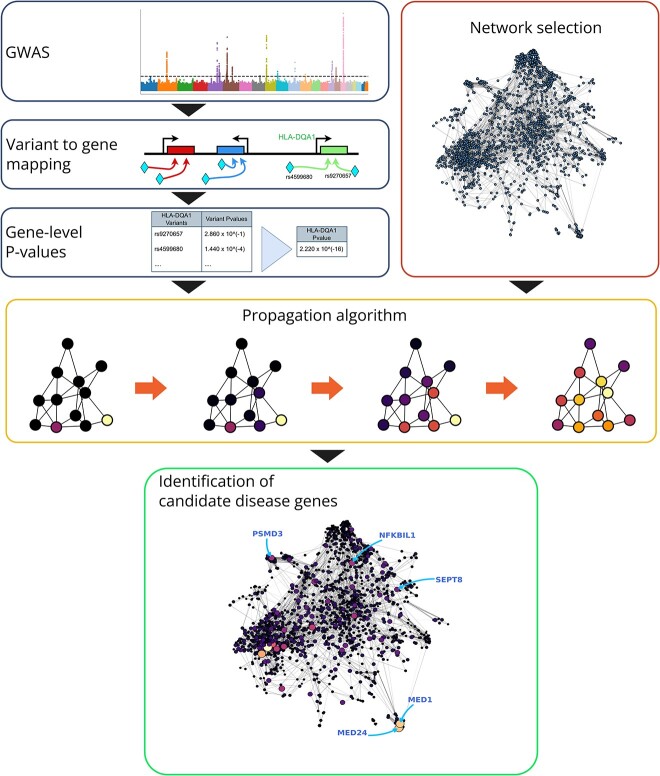
Overview of the workflow for the application of network propagation methods to GWAS summary statistics. The analysis of GWAS summary statistics begins with the selection of a methodology to map variants to protein-coding genes. The *P*-values for the variants associated with each gene are aggregated to generate gene-level *P*-values. The scores are overlaid on a selected molecular network, and the information is diffused with a suitable propagation algorithm. A selection criterion is then employed to obtain sets of candidate disease genes from the propagated information. Each of the steps involved presents important design choices that affect the results obtained through this approach.

## BACKGROUND

The procedure for GWAS consists of a number of crucial experimental and computational steps, including the selection of appropriate study populations, data collection, genotyping, imputation, quality control, association testing and replication. These steps are reviewed in detail in [9]. Here, we focus on the downstream analysis that starts with the mapping of variants to genes, and the aggregation of the variant *P*-values to gene-level scores. It is followed by selecting the network and the propagation algorithm. Lastly, putative disease genes are identified based on ranking or similarity criteria.

### Mapping genetic variants to genes

The first step is mapping the scores of individual variants for association with the disease (generally *P*-values) to a corresponding gene [10]. Here, we focus on SNPs, as they are the most common type of genetic variant analysed in GWAS, although the following discussion applies to other small-scale variants.

Associating SNPs to genes can be done in three main ways. The simplest approach is to associate with each gene the SNPs that fall within the start and end of the gene body. Since many significant disease-associated variants fall into intergenic regions [11], the gene borders are generally extended with buffers of several thousand base pairs to account for nearby promoter or enhancer elements connected to the gene. However, distal trans-regulatory elements such as enhancers can often be found at high genomic distances [12]. These important functional elements far outnumber the genes in the human genome [13]; additionally, enhancers may have no functional effect on proximally located genes, but regulate far away genes instead. The simple genomic distance method can therefore lead to missing or spurious associations between loci and genes.

An alternative methodology is *chromatin interaction mapping*, in which we consider physical distances in 3D chromatin contact maps [14] and associate SNPs with the nearest gene within each topologically associated domain (TAD)[7]. TADs are segments of the genome that are highly enriched for DNA-DNA contacts within the segment; owing to this topological constraint, TADs are thought to group variants with the genes they likely regulate.

Finally, SNPs, in particular variants in non-coding regions, can be associated with genes by examining their correlation with gene expression level as phenotype. SNPs with this effect are called *expression Quantitative Trait Loci* (eQTL). eQTLs offer great functional insight on the relationship between SNP loci and the genes they may regulate; however, they show considerable variation across tissues, which may impose additional challenges for their use in the network propagation framework. A careful selection of eQTLs in tissues that are relevant to the disease studied is, therefore, a crucial element for the robust application of this method.

Comparisons between these three methods for SNP-gene mapping have shown that the choice of methodology has a considerable impact on the number of gene-SNP associations considered in the analysis [15]; the selection of an appropriate mapping approach is an experimental design choice that requires evaluation for its impact on downstream analysis.

### Generating gene-level scores

Once each SNP is associated with a gene, their individual scores have to be combined into a gene-level score. The most straightforward approach is to assign to each gene a binary value indicating the presence of SNPs that show significant association with the disease. However, previous works have shown that the use of network propagation for signal amplification is hindered when using discrete values [16].

The alternative approach is to combine the *P*-values for SNPs into an aggregate gene-level *P*-value. This score generally outperforms the discrete values, possibly because it transfers more information from GWA studies by assigning larger weights to genes with the strongest associations [10].

Among the aggregation methods, the simplest is to assign to each gene the lowest *P*-value (i.e. the strongest association) within the SNPs mapped to the gene (an approach often referred to as minSNP) [17, 18]. This approach is biased towards longer genes, and does not account for LD between SNPs. To overcome this bias, subsequent methods included either permutations [19, 20], or simulations (VEGAS [21], VEGAS2 [22]) of the SNPs associations. Such algorithms tend to require access to the underlying genotypes and are computationally intensive.

A more efficient methodology is the use of regression-based models (SKAT [23], MAGMA [24]), which can include covariates to account for population stratification. Nevertheless, these methods still require access to genotypic data, which is inconvenient when working with GWAS summary statistics.

A few aggregation methods include additional information to improve the statistical power of the downstream analysis, such as GATES [25], that can assign weights to SNPs based on functional information, or RAREMETALS [26], which adopts a meta-analysis framework. More recently proposed, COMBAT [27] is an ensemble method that combines VEGAS, GATES and SimpleM, a variant of minSNP that includes multiple hypothesis correction [28].

Another interesting approach, although less used, is the circular genomic permutation (CGP) method [29]. CGP can be applied to GWAS summary statistics, corrects for the gene length bias and considers the LD between SNPs. Furthermore, An exact and efficient algorithm for CGP, called fastCGP, has been recently proposed [30].

One of the more recent works that focuses specifically on the aggregation of *P*-values is PEGASUS [31], a method that computes gene scores analytically from a null chi-square distribution that captures LD between SNPs in a gene. It requires only GWAS summary statistics and a reference population for the LD calculations. PEGASUS is not biased to gene length and is sensitive to genes with multiple SNPs that are moderately associated with the phenotype of interest. A similar method that makes use of efficient numerical approximations for an analogous test statistic is fastBAT [32].

Network propagation methods that leverage such gene-level *P*-values tend to assign to each node the $-log_{10}(\text{P-value})$ score.

### Selecting the network

The diverse biological and functional relationships between genes and gene products are captured in molecular networks [33]. In a *molecular network*, nodes represent biological entities (e.g. genes, proteins, metabolites), and the edges represent direct or indirect relationships. In a protein–protein interaction (PPI) network, for example, the proteins represented by the nodes are connected if they share a functional relationship. The label ‘molecular network’ includes PPI networks, gene co-expression networks, metabolic networks or gene–gene Interactions networks.

In the analysis of disease genes, PPI networks are the most commonly used molecular networks because proteins perform a variety of critical functions in the biological processes that sustain an organism [34]. However, PPI networks are incomplete and biased towards extensively studied genes [35, 36]. It has been observed that the limitations of existing PPI networks may constitute the most significant bottleneck to the use of network propagation methods for GWAS data [16]. Several methods have been developed to compensate for the limitations of PPI networks, with approaches such as correcting for node degree (DADA [37]) or ‘coreness’ (NetCore [38]).

Various algorithms have been proposed to reduce noise in molecular networks in general. For example, DRaWR [39] adopts a two-stage RW method, where a first propagation cycle extracts a relevant subnetwork from a heterogeneous graph, to then apply a Random Walk with Restarts (RWR) algorithm for gene ranking.

A recent systematic evaluation of molecular networks offered insights on their use for the identification of disease genes [40]. Generally, performance scales with the size of the network; while more extensive networks contain more false positives, it appears that the discovery of new interactions outweighs this issue [40].

### Propagation algorithms: mathematical formalisms

The term *network propagation* is a broad categorization that encompasses various methods. Generally, a network propagation method superimposes some information on a graph and then iteratively diffuses this information to highlight nodes (genes) related to the original signal. The specifics of this process can vary significantly depending on the diffusion mechanism modelled (e.g. RWs on graphs, heat diffusion), the type of graph (directed or undirected), the number of graphs, and other design parameters. A comprehensive review of the general use of network propagation for disease genes discovery can be found in [8].

RWs on graphs offer an efficient formalism to model the transfer of information. We can represent an undirected graph using a *normalized adjacency matrix* W, and the measured gene-level scores as a vector $p_{0}$. With this structure, an iteration of the information diffusion process can be calculated as 


(1) \begin{align*}& p_{t} = W p_{t-1}\end{align*}

Indefinite repetition of the diffusion step ends up in uninformative states where all nodes have uniform value; because of this, most of the network diffusion methods that explicitly model RWs employ a variant named RWRs, also known as personalized PageRank (a variant of PageRank [41]) or insulated diffusion. In an RWR framework, in addition to the standard transition probabilities defined by the adjacency matrix, each walk has a certain probability of returning to the root node. In suitable conditions (connected network and eigenvalues of W not larger than 1), this process converges to a steady-state that can be calculated as 


(2) \begin{align*}& p_{s} = (1-\alpha) \left(I - \alpha W\right)^{-1}p_{0}\end{align*}

where $\alpha $ is a parameter that regulates the trade-off between diffusion and retaining the initial information, by controlling the probability of continuing the walk at each step.

In general, efficient propagation methods can be calculated using matrix multiplication between the initial state $p_{0}$ and a suitable matrix $W_{\textrm{graph}}$ derived from the adjacency or Laplacian matrices of the graph.

This formalism can be extended to account for directed or weighted graphs, multiplex and heterogeneous networks, and more.

Network propagation is a special case of graph convolutions, a mathematical operation extensively used in graph neural networks, which would suggest the possibility of extending the information diffusion analysis using a deep learning framework. However, due to the low power of GWA studies, deep learning models run the risk of overfitting and being outperformed by more straightforward propagation methods.

### Identifying potential risk genes based on rank or similarity

Network propagation methods can be divided into two main categories, depending on the desired output information.

*Ranking-based* methods aim to assign a score to each gene in the network and then rank their significance based on this score. Within this framework, an important design choice is the selection of significant genes based on the final scores. The simplest approach is to fix a hyperparameter $N$ and select the top *N* genes in the ranking. Alternatively, methods that propagate *P*-value scores can use FDR control to select a threshold or distribution inflexion point thresholds.

*Similarity-based* methods evaluate gene-gene similarity to detect dense gene modules. Such methods build similarity matrices between genes and then look for clustered submatrices that show high internal similarity. The general underlying assumption is that densely connected subgraphs in a molecular network include genes working for common functionality, which, when altered, can be the causal factor for a disease.

The selection of disease genes using scores derived from network propagation offers interesting opportunities that can complement more domain-focused methods and expand the pool of candidate disease genes. Alternative computational methodologies for the identification of disease genes often rely on knowledge databases such as Gene Ontology [42] and KEGG [43]. Such domain- or pathway-based approaches often suffer from considerable biases towards prior knowledge [44]. Network propagation methods, while not free from biases, offer a complementary approach that enables an alternative search strategy for the exploration of possible disease genes.

### Beyond a single undirected graph

The basic formulation of network propagation needs only a single undirected graph. However, we may wish to include more domain knowledge in our analysis, which can be accomplished in a variety of methods.

A simple extension includes the use of multiple graphs with an ensemble method. The initial scores can be propagated separately on each graph, and the resulting scores can be merged for unified analysis. The combination of scores can be a simple average of the ranks on individual networks or a more elaborate combination that is useful for multiple tasks, such as the low-dimensional representations produced by Mashup [45].

Alternatively, multiple graphs can be combined in a single propagation model by using RWs on multilayer or multiplex networks [46, 47]. At each step, the walk can proceed to neighbouring nodes in the same network or to the corresponding node in a different network.

Finally, the diffusion formalism can be extended to heterogeneous networks, i.e. graphs whose nodes can represent different types of entities (e.g., gene-phenotype networks). Heterogeneous networks capture the interaction between various biological entities in a manner that multiplex graphs cannot emulate. An example of this approach is the bipartite graph propagation in [48], where one set of nodes representing individuals is connected to a second set representing relevant SNPs.

Any combination of the previous extensions is feasible to include as much domain knowledge as possible. RWRs on heterogeneous multiplex networks, for example, have shown great promise in combining different types of interactions for the identification of disease genes [46].

### Network propagation for identification of disease genes

The most widespread approach for identifying putative causal disease genes is to select previously known disease genes (e.g. those deemed as significantly associated with the disease by GWAS) that are used as starting ‘seeds’ to propagate information, often using PPI networks. RWRs and heat diffusion models are the most common formalisms adopted for the actual propagation step, possibly with additional processing steps to reduce the FDR and limit the biases in the molecular graphs.

Overall, RWRs are the basis for most state-of-the-art methods, generally including additional measures to improve power or robustness. Such measures include the use of heterogeneous networks [49], weighted PPI networks and estimates of prior probabilities (PRINCE [50]), or reweighting of interactions close to known disease genes (ORIENT [51]).

Another possible improvement to the RWR methods is to change the simple ranking of resulting gene scores to a more elaborate comparison scheme. DP-LCC [52], for example, compares diffusion profiles (i.e. the stationary distributions obtained from RWRs) for known disease genes and the candidate disease genes considered in the analysis. Candidate genes are then prioritized according to the similarity of their diffusion profile with the query disease genes.

One of the most promising directions of development is the inclusion of additional sources of information to the analysis process. Several methods, for example, include information on disease genes from closely related diseases (NPDE [53]), based on the assumption that such diseases are likely to have a similar molecular basis [54]. The use of multi-omics datasets represents another active area of research; network propagation methods can make use of these transversal sources of information either by separately analysing each channel of information and then combining their results, or by propagating a vector representation of each node in the network, where each feature encodes the information from one dataset for that gene [55]. A potential source of this kind of complementary information is represented by the significant gene-trait associations derived from Transcriptome-wide Association Studies (TWAS) [56, 57]. TWAS have demonstrated good power in the presence of pleiotropy and higher heritability of expression [58]. Gene expression and its genetic regulation are central focus points for TWAS and, although some works have criticised their monolithic use for both feature selection and aggregation [59], they have demonstrated remarkable efficacy in detecting disease-gene associations and offering insights into the mechanisms of these links [56].

Finally, the aggregation of signals across possible disease genes for the identification of significant subnetworks offers exciting opportunities for improving the statistical power of the analysis performed. For the identification of gene modules, some of the most popular models include HotNet [60], and its derivative versions HotNet2 [61], and Hierarchical HotNet [62], that model an insulated heat diffusion process. The advantage of HotNet and other similarity-based methods is that they combine the signals of rare variations that affect the same biological processes, thus considerably improving statistical power. In contrast, the individual SNPs might be missed by a simpler ranking-based propagation method.

## METHODS

In the following sections, we present a set of benchmarking experiments that we use to highlight and corroborate general trends that emerge from the literature on network propagation for disease gene discovery. We describe here the sources of data used for the selected diseases and molecular networks, and the evaluation metrics used. Finally, we present the results observed in this case study.

### Data

GWAS summary statistics were selected for three diseases as case study, specifically asthma [63], autism spectrum disorder (ASD) [64] and schizophrenia from [65]. Data for ASD and Schizophrenia data have been downloaded from the Psychiatric Genomics Consortium^1^ . Data used for asthma are available through the GWAS Catalog [66].

The three sets of summary statistics were processed using PEGASUS [31] with the provided additional files derived from the 1000 Genomes project.

Target genes for the three diseases were derived from the GWAS Catalog [66] (downloaded on 26 January 2023). The data downloaded also include the genes associated with each SNP (“Mapped gene”); all genes included in the set of SNP associated with the target disease with a *P*-value $\leq 5 \times 10^{-8}$ were gathered as the target set.

Table 1 reports the number of seed and target genes resulting from the preprocessing of the GWAS summary statistics.

**Table 1:** For each disease, we report the number of genes selected as seeds based on their aggregate *P*-value resulting from PEGASUS, as well as the number of target genes that do not overlap with the set of seed genes.

Disease	N. seed genes	N. target genes
Asthma	118	625
ASD	30	723
Schizophrenia	536	949

### Gene networks

For the RWR experiments, we selected five molecular networks covering several facets of gene biology, which were downloaded from NDEx^2^ [67–69]. HumanNet (v3) [70] combines protein-protein interactions and gene functional information. We specifically selected HumanNet-XC, a version of the network extended by co-citation. BioPlex 3 [71] consists of two PPI networks derived from 293T (human embryonic kidney) and HCT116 (colorectal carcinoma) cell lines. We selected the network composed of the shared interactions between the cell lines, which represents conserved mechanisms in human biology. ProteomeHD [72] is a co-regulation map of the human proteome. We downloaded the smaller version of the network, which covers the top $0.5\%$ co-regulated proteins. PCNet (Parsimonious Composite Network) [40] is derived from merging several molecular networks and preserving interactions supported by at least two networks. STRING [73] results from combining information from high-throughput experimental data, literature and database mining, and genomic context analysis.

These networks were selected to test the performance of network propagation in a variety of settings. STRING, for example, is a well-established network in the domain of PPI analysis and, like HumanNet, it aggregates information from several sources. BioPlex3 and ProteomeHD, instead, represent networks derived from a more selective analysis, and offer test cases with a smaller number of vertices than the aforementioned networks. PCNet, finally, is a composite network explicitly derived from a systematic evaluation of network analysis for disease gene discovery; for this reason, we wished to examine whether this purpose-made network could outperform networks that are not specifically designed for this task. When joined into a multilayer network, these molecular networks offer complementary views of the interactions between genes, which ideally enhance the outcome of a network propagation analysis.

In Table 2, we report the size of each network.

**Table 2:** Sizes of the networks analysed.

Network	N. nodes	N. edges
HumanNet v3 [70]	18 593	1 125 494
BioPlex 3 [71]	8364	35 704
ProteomeHD [72]	2718	63 290
PCNet [40]	20 517	2 693 250
STRING [73]	17 079	420 534

### Performance metrics

Evaluating disease gene prediction models is far from trivial. Several methods in the literature adopt a leave-one-out methodology: considering a set of disease genes, one of them is removed, while the rest is fed as input to the model, to then evaluate how well the target gene is retrieved. This methodology is convenient, but it risks introducing selection biases in the model, as they are tuned to perform well on this specific task rather than how well it discovers new disease genes in general.

To overcome this limitation, our testing setting considers only data from a GWA study performed before the year 2020, while targeting all the currently known disease genes found in GWAS analyses. We essentially aim to answer the question: *how well does the algorithm rank disease genes that were discovered by subsequent studies?*

This setting is similar to many common information retrieval tasks. We therefore measured the prediction performance using the standard metric *Average Precision@K* (AP@K), an ordered ranking metric that evaluates the relevance of the top K predictions, as well as their position in the ranking.

Other works in the literature have measured the performance of network propagation analyses through the use of classification metrics such as the area under the receiver operating characteristic curve [74]. We believe, however, that an ordered ranking metric such as AP@K offers a more complete picture of the effectiveness of an algorithm in ranking putative disease genes so that the most promising candidates appear at the top of the ranking.

AP@K is derived from Precision@K (P@K), which measures how many of the first K items predicted are actually relevant: 


(3) \begin{align*}& P@K = \frac{\text{N. of relevant genes in the first K ranks}}{K}\end{align*}

Average Precision@K is the average of P@N for the values of *N* ranging from 1 to K that correspond to relevant items. Several definitions of AP@K are found in the literature. We adopt the version of AP@K in which the normalization factor is defined as the minimum of K, and the total number of relevant items M, which ensures that the AP@K always ranges between 0 and 1, for all queries [75]: 


(4) \begin{align*}& AP@K = \frac{1}{min(K, M)} \sum_{N=1}^{K} P@N * rel(N)\end{align*}

where $rel(N)$ is a binary indicator variable that assumes the value 1 if the *N*th ranked gene is a true positive, and 0 otherwise.

In our experimental setting, AP@K captures the number of relevant disease genes retrieved by the propagation algorithm, favouring configurations that rank the relevant genes higher among the top K predicted.

The averaging of AP@K over all possible disease-network queries $(dis, net)$ is used to calculate the *mean Average Precision@K* metric, which offers a global summary of the performance of network propagation methods: 


(5) \begin{align*}& mAP@K = \frac{1}{|(dis, net)|} \sum_{(dis, net)} AP@K(dis, net)\end{align*}

## RESULTS

Several recent works have included benchmarking and evaluation of propagation methods [10, 34, 49, 74].

Determining the effectiveness of a network propagation algorithm is quite challenging. The approaches chosen include leave-one-out validation and external validation with selected datasets. We note, however, that since we often lack access to ‘gold standard’ datasets, any such evaluation methodology risks including systematic biases that would reward models that detect known and well-studied disease genes, while penalizing models that classify rare variants as significant.

The development of a standard set of benchmarking tasks for the evaluation of network methods would be highly beneficial for robust comparisons between state-of-the-art models. Nevertheless, such a task is quite daunting and would require the selection of diseases where disease genes are known with high certainty and possibly the use of simulated sets.

We present here a set of benchmarking experiments to analyse some design choices involved in applications of network propagation. We simulate a strict experimental setting, where the only input data available comes in the form of summary statistics of GWA studies. Several methods in the literature, such as PRINCE [50] and VAVIEN [76], include information on disease genes from diseases related to the target disease that is being analysed. We forego this approach to evaluate the basic factors contributing to the performance of network propagation methods.

As disease case studies, we select asthma, ASD and schizophrenia. For each disease, we downloaded GWAS summary statistics from a publicly available study, and processed them using PEGASUS [31] to generate gene-level *P*-values. While more recent frameworks for the analysis of GWAS *P*-values have been proposed, they do not focus specifically on the aggregation of gene-level *P*-values like PEGASUS, which was therefore employed.

We define as *seed genes* the genes that result statistically significant from the aggregated SNP *P*-values. To select the seed genes, we use a nominal significance level of 1%, which was divided by the number of genes present in the processed data for a given study to get a Bonferroni-corrected *P*-value; the significance level and the multiple hypothesis testing correction are chosen to provide a stringent selection criterion that limits the inclusion of false positives in the pool of disease genes. The genes whose aggregate *P*-values fall below the adjusted *P*-value threshold are considered significant. In our experimental setting, the seed genes represent the prior knowledge, i.e. the genes for which we are sufficiently certain that a link to the disease analysed exists.

We define as *target genes* all the genes that are currently known to be relevant for the diseases considered. Target disease genes are derived from the GWAS Catalog [66], by selecting all known SNPs associated with the target disease with a *P*-value $<5*10^{-8}$ and collecting all the associated genes. We remark that the association of a gene with a significant SNP in the GWAS Catalog does not necessarily describe the causal role of the gene for a disease. Further annotation and functional analysis is needed to draw such conclusions. However, these target genes provide a convenient measure to determine how well the network propagation experiments detect relevant genes.

The genes at the intersection of seed genes and target genes for a disease are removed from the pool of targets, to ensure only new knowledge is considered in the evaluation of performance. Essentially, the experimental question tested involves measuring how many of the currently known disease genes we can retrieve, starting from the incomplete knowledge provided by a single GWAS.

Five gene networks have been downloaded for testing network propagation algorithms: PCNet [40], BioPlex3 [71], ProteomeHD [72], HumanNet v3 [70] and STRING [73].

To measure how well each network propagation experiment manages to retrieve target genes that do not appear as significant in the original GWAS summary, we employ the standard information retrieval metric AP@K, which accounts for both the number of correct predictions in the top K genes and for their position in the ranking. AP@K can be aggregated over all disease-network queries using *mean Average Precision@K* (mAP@K), which provides a convenient summary of the performance of network propagation approaches. All the experiments are performed with the PageRank implementation of the RWR algorithm from the python package *NetworkX* [77].

### Gene scores outperform seed genes for conservative RWRs

Most network-based methods for disease gene identification in the literature make use of gene seeds, known disease genes that are used as a starting point to find new candidate disease genes. While well established, this approach assigns equal importance to all disease genes, which may not reflect their role in causing the disease.

Assigning different scores to each gene would allow more flexibility in this regard. Particularly in the case of GWAS analysis, we can derive gene-level *P*-values and assign to each gene as score the negative logarithm of the *P*-value ($-log_{10}(\textit{P}\text{-value})$). In this way, more significant genes are given a higher weight in the propagation procedure.

We compared the performance of RWRs with different selections of the parameter $\alpha $, which represents the probability of continuing the RW at each node, and selecting the K top ranking genes as candidates.

**Figure 2:**
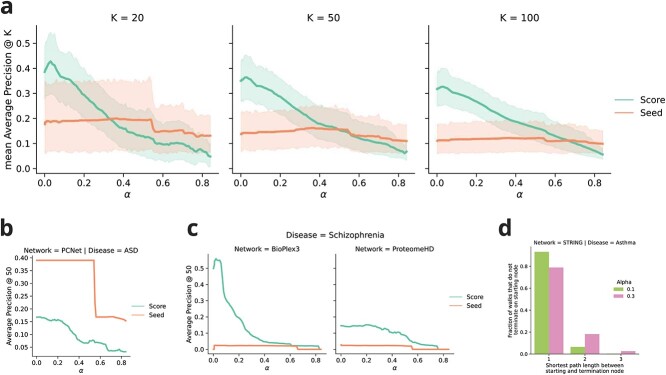
Comparison of RWRs based on gene-level *P*-values versus the use of seed genes. (**A**) Range of performance across diseases and networks for different values of the probability of continuing the RW. The use of seed genes is more robust to hyperparameter tuning, but gene scores outperform seed genes when selecting $\alpha $ close to 0. The shaded areas represent the 95% confidence interval. (**B**) The combination ASD-PCNet proves to be the only outlier where the use of seed genes clearly outperforms the *P*-value-based scores. (**C**) The performance of the RWRs shows considerable variability across disease-network combination. The ProteomeHD network, in particular, shows poor performance, likely due to the small number of available genes. (**D**) Results of the simulation of RWRs starting from asthma seed genes using the STRING network, for two example values of $\alpha $. For each gene, we performed RWRs for 100 000 restarts, and considered the shortest path distances between the starting gene and the termination gene, filtering out the walks that terminate on the starting gene. The resulting histogram shows the fraction of walks that terminate on nodes that are one, two or three hops away from the starting gene. This distribution can be used as a criterion to select the hyperparameter $\alpha $ based on the desired level of exploration of the network.

In Figure 2(A), we compared the performance of network propagation with the use of gene scores versus using the seed genes to assign an initial binary value to the nodes. The plot displays the mean of Average Precision at K (i.e. mAP@K) for the retrieval of target genes among the top 20, 50 and 100 genes in the rankings across all network-disease combinations. The value of the plotted line at $\alpha = 0$ corresponds to skipping the propagation step, and was omitted for the seed genes method, as the AP@K would be 0 by our definition of the prediction target.

It is clear that the use of *P*-value-based scores preserves much more information and allows us to retrieve more relevant genes than simply assigning a binary value to indicate significance. In all but one of the disease-network combinations, the *P*-value scores outperformed the use of seed genes, with the notable outlier represented by the combination ASD-PCNet (Figure 2(A) and 2(C)).

We also highlight a general increase in performance with a conservative RWR process ($\alpha $ close to 0) compared to skipping the propagation step ($\alpha =0$). In 12 out of 15 of the disease-network combinations, the highest AP@K is achieved with a positive value of $\alpha $ (i.e. with the contribution of the propagation step), corroborating the usefulness of diffusion methods. However, the trend shows high variability across the tested configurations, as shown in the example in Figure 2(C).

The improvements found for small values of $\alpha $ suggest that the local neighbourhood of disease genes is more important than the global properties of the graph for the identification of disease genes. This observation is consistent with the established literature; some network-based methods, such as ORIENT [51], have been explicitly designed to exploit this fact.

An additional observation to consider is that the best performing network is not constant across diseases. Using the score-based propagation, HumanNet achieves the highest AP@50 for asthma and ASD, while PCNet performs better in the analysis of the schizophrenia GWAS. This result is consistent with previous works in the literature, which highlighted how the choice of molecular network can have a large impact in the analysis of certain diseases [40].

The choice of the $\alpha $ parameter in a practical setting would depend on the desired outcome of downstream analysis. Some works in the literature framed this parameter optimisation within the framework of the bias-variance trade-off and aimed to tune the parameter using consistency across replicates and across -omics datasets [78]. Alternatively, the $\alpha $ parameter could be tuned to control how far to range from known disease genes. As an example of this kind of procedure, we considered the STRING network and the set of seed genes related to asthma. Starting from each seed gene, we performed RWRs with two different $\alpha $ parameters, for 100 000 restarts each. For each termination node (i.e. where the walk restarts) of the RWs, we calculated the shortest distance path from the starting disease gene, filtering out the walks where the start and the termination correspond. In Figure 2(D), we show the fractions of such random walks that end up on genes that are one, two or three hops away from the starting gene. The RWRs with $\alpha = 0.1$ are essentially influenced only by the one-hop neighbourhood of the disease genes, while $\alpha = 0.3$ shows influence from the two- and even three-hop genes. Using a similar procedure, a researcher may tune the restart probability to select the desired level of exploration of the network.

### Larger networks display better performance

**Figure 3:**
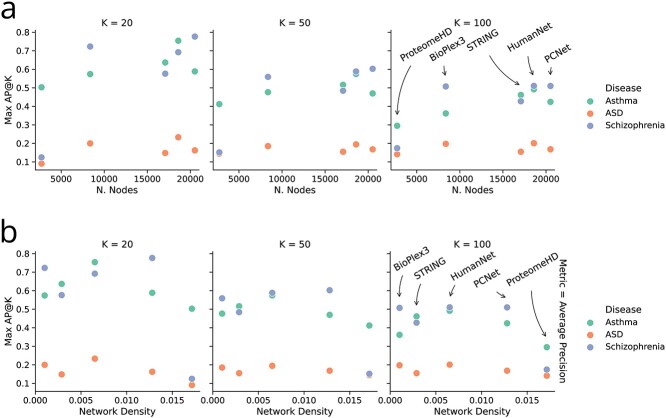
Network properties affect the result of propagation algorithms. (**A**) Best performance of the network propagation method for each network, plotted against network size. An upward trend is apparent, suggesting that larger networks are beneficial for this analysis. (**B**) The performance of the network propagation methods for different densities of connections displays a peculiar pattern, suggesting that networks that are too sparse or that include too many connections may hinder the use of network propagation.

Previous works have suggested how the size of the network may play an important role in the performance of network-based analysis of disease genes [40, 74]. We visualise this dependency in Figure 3(A), where we selected the best performing RWR for each network and each disease. The scatter plots display a general pattern of improved performance with larger networks. This trend is likely rooted in a multitude of causes, not least of which the fact that smaller networks may not include the highly significant genes from the GWA study or important target genes (as clearly highlighted by the poor performances of the ProteomeHD network, the smallest of the molecular networks considered).

Notably, the performance of the propagation procedure is quite low on the data for ASD, possibly due to the lower statistical power of the available summary statistics.

In addition to the size, the connectivity of the network may contribute to determining its relevance to the disease analysed. We measured the network density, defined as the ratio of observed edges to possible edges, which we can represent as 


(6) \begin{align*}& D_{net} = \frac{N_{edges}}{N * (N-1)/2}\end{align*}

where *N* is the number of nodes in the network.

Figure 3(B) presents the best-performing RWR for each network and each disease.

The trend presented suggests that a graph that is neither too sparse nor too dense would be the best candidate for network propagation methods. Most likely, sparse networks lack the necessary connections to transfer information to relevant disease genes, while dense networks allow the information to diffuse too much to isolate promising candidates. This result emphasises the need to expand known gene-gene networks, but always keeping the rate of false positives into account.

### Ensemble methods can improve on the use of a single network

The use of a single network is convenient from a computational perspective, but may restrict the information on gene-gene relationships that we can use in our analysis. We tested how an ensemble derived from the RWRs on a single network performs in the task of retrieving disease genes. Specifically, we considered the rankings generated by averaging across networks the rank of each gene (Avg. Rank), provided their presence in at least two networks.

Additionally, we performed RWRs on the multilayer network generated by connecting all 5 networks. To perform these experiments, we consider the adjacency matrices of each network, and join them into a single overarching matrix. We then add to this global adjacency matrix the edges that connect the same gene in different networks. The result is the multilayer *supra-adjacency matrix* [79], which can be used to extend the RWR formalism to multilayer graphs [47].

**Figure 4:**
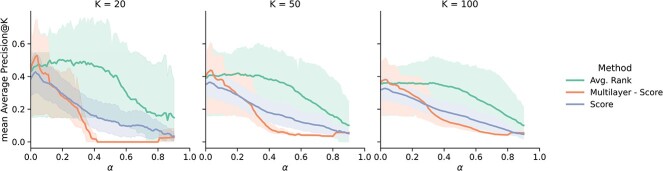
Comparison of the performance for single network RWRs to the two listed Avg methods and RWRs on a multilayer network composed by the five gene networks, represented as mAP@K with 95% confidence interval. *Avg. Rank* appears to offer robust performance even for high values of $\alpha $, which enables the explorations of genes further apart from known disease genes.

The performance of these methods is compared to the range of performance for single network RWRs for each disease in Figure 4.

Generally, *Avg. Rank* outperforms the use of single networks, while the Multilayer RWR performs well only for very localized RWs. For the latter, we hypothesize that connections to the denser networks allow the information to easily diffuse away from disease genes quicker than single network propagation for increasing values of $\alpha $, which results in a loss of information. Genes that rank high on multiple individual networks, on the other hand, are likely to be more relevant to the target disease.

*Avg. Rank* also appears to offer robust performance for higher values of $\alpha $, which corresponds to the case where information can diffuse further from the starting configuration. This approach, therefore, has the potential to explore disease genes that are not found in the immediate neighbourhood of known disease genes, but that are reached by traversing more edges in the graph.

## CONCLUSIONS

The use of molecular networks to improve the power of GWAS analysis is a promising tool that requires several design choices to be most effective. Firstly, we must select one or more appropriate networks; for this step, a general rule of thumb seems to be to include as much information possible (i.e. more extensive networks and more graphs) with the secondary aim of limiting the number of false positives in the interactions. PPI networks are widespread in the literature. However, in the case of GWAS, it is common to find SNPs for genes that are not well studied in PPI graphs. Several works have shown great promise with heterogeneous networks, although the node types should be selected to be relevant for the disease analysed.

Afterwards, the SNPs in the GWAS study must be mapped to the relevant genes to combine SNP-level *P*-values into gene-level scores. Several frameworks have been developed to this end, with recent development offering more efficient methodologies that require only linkage disequilibrium data in addition to the GWAS summary statistics.

The choice of propagation algorithm depends on the desired outcome for the selection of putative causal genes. Similarity-based methods seem to offer a competitive edge over ranking-based methods regarding statistical power. However, ranking methods may offer improved performance for loosely connected causal genes, although the choices of specific algorithms would vary greatly depending on the network used.

In general, the design choices for each step of the network propagation procedure also depend on the downstream analysis that a researcher might want to implement. The most direct application is to simply perform a network propagation analysis, and then select the *N* genes with the highest-ranking scores that are not currently listed among the known disease genes for the target disease. This pool of candidates can then be validated by experimental work or other sources of biological evidence. Another possibility would be to use network propagation as a principled way to examine the similarity of diseases: if overlapping sets of genes score high in the propagation procedure from two diseases, it might indicate a deeper connection between the underlying mechanisms.

We performed benchmarking experiments in a strict setting where the only data available comes in the form of GWAS summary statistics. RWRs performed using five selected networks revealed that the use of gene scores (i.e. $-log_{10}(\textit{P}\textit{-value}_{gene})$) is better able to retrieve currently known disease genes from the GWAS Catalog when using RWRs that focus on the neighbourhood of known disease genes. The size of the networks appears to be positively correlated with the performance, consistently with other works in the literature. However, analysing the network density suggests that both insufficient and excessive connectivity may negatively affect the propagation process.

Finally, we tested two methodologies to combine multiple networks, one of which (Avg. Rank) outperformed the other methods in several settings. Additionally, the averaging method displayed good performance even for expansive RWs with restarts (i.e. RWRs that range further from the originating node rather than restarting frequently), which could enable the discovery of putative disease genes that are not directly connected to known disease genes in the available molecular networks. This result suggests that careful inclusion of multiple networks is beneficial to improve the power of GWAS analysis.

The specific networks and diseases analysed in this case study offer us insights into the challenges and benefits of using network propagation for identifying candidate disease genes. This framework, however, offers great flexibility, as it can be expanded by including additional sources of information and more complex propagation procedures. Overall, we believe that network propagation constitutes a valuable tool to employ for expanding the pool of candidate disease genes for a variety of diseases.

Future works might examine extensive criteria for the selection of one or more networks based on disease-specific criteria. Additionally, the combination of different sources of information with GWAS summary statistics is a promising research direction that is likely to offer more in-depth insights into the biological mechanisms of disease.

**Key Points**

- Molecular networks offer a complementary layer of information that can improve the analysis of GWAS summary statistics.
- Network propagation methods model the diffusion of information using molecular networks. These approaches enable the identification of candidate disease genes or gene modules that would otherwise be missed with a direct statistical analysis of GWAS results.
- The use of *P*-values to assign scores to the genes represented in the network offers more robust performance for the identification of putative disease genes.
- The specific network and its properties, the propagation algorithm, and the scoring criterion constitute important design choices for the implementation of network propagation approaches.
- Ensemble methods may improve the effectiveness of network propagation, and enable the identification of possible disease genes outside the immediate neighbourhood of known disease genes.

## Data Availability

The datasets were derived from sources in the public domain. Specifically, GWAS summary statistics were obtained from the Psychiatric Genomics Consortium^3^ and the GWAS Catalog^4^ [66]. Known disease genes are also derived from the GWAS Catalog. The molecular networks are downloaded from NDEx^5^ [67–69].

## Footnotes

1
https://www.med.unc.edu/pgc

2
https://www.ndexbio.org

## CODE AVAILABILITY

The code used to generate the results presented in this work can be found at https://github.com/gvisona/GWAS_NetworkPropagation.

## Author Biographies

**Giovanni Visonà** is a PhD student at the Max-Planck Institute for Intelligent Systems in Tübingen, Germany. He is an ESR in the Marie Curie Innovative Training Network entitled ‘Machine Learning Frontiers in Precision Medicine’.

**Emmanuelle Bouzigon** is a senior Researcher at the French National Institute of Health and Medical Research (INSERM) and GEMS (Genomic Epidemiology of Multifactorial diseaseS) Group Co-Leader at the Inserm-Université Paris Cité UMRS-1124 unit, Paris, France.

**Florence Demenais** is the director of Research Emeritus at the French National Institute of Health and Medical Research (INSERM) and GEMS (Genomic Epidemiology of Multifactorial diseaseS) Group Leader at the Inserm-Université Paris Cité UMRS-1124 unit, Paris, France

**Gabriele Schweikert** is a principal investigator for the Division of Computational Biology (School of Life Sciences) of the University of Dundee in Dundee, Scotland, UK.
